# In the End: Associations Between Sleep Disturbance and Functional Impairment in Fibromyalgia—A Path Analysis Study

**DOI:** 10.1111/jsr.70199

**Published:** 2025-09-17

**Authors:** Kristoffer Bothelius, Eva Kosek, Markus Jansson‐Fröjmark

**Affiliations:** ^1^ Clinical Pain Research, Department of Surgical Sciences Uppsala University Uppsala Sweden; ^2^ Department of Clinical Neuroscience Karolinska Institutet Stockholm Sweden; ^3^ Centre for Psychiatry Research, Department of Clinical Neuroscience Karolinska Institutet, & Stockholm Health Care Services, Region Stockholm Stockholm Sweden

**Keywords:** chronic widespread pain, cognitive biases, disability, fibromyalgia, mood disorders, psychological distress, sleep quality

## Abstract

Fibromyalgia is a chronic pain condition characterised by widespread pain, sleep disturbances and mood disorders, often leading to significant functional impairment. Although sleep problems are recognised as important contributors to fibromyalgia symptoms, the mechanisms linking sleep disturbances, psychological factors and functional impairment remain insufficiently understood. This study investigated the direct and indirect relationships among sleep disturbances, pain catastrophising, depression, bodily pain and functional impairment using path analysis. Data were drawn from 253 women diagnosed with fibromyalgia. Poor sleep quality was directly associated with elevated levels of pain catastrophising and depression, and both psychological factors were, in turn, significantly related to greater functional impairment. Together, these variables accounted for 32.5% of the variance in functional impairment. No significant indirect effects were observed between sleep disturbances and functional impairment through bodily pain, and pain severity was not significantly associated with functional outcomes. These findings underscore the critical role of poor sleep quality in functional impairment among individuals with fibromyalgia. Implementing interventions that target and improve sleep may, in turn, alleviate related psychological distress, reduce disability and enhance quality of life in this population.

## 1 Introduction

**FIGURE 1:**
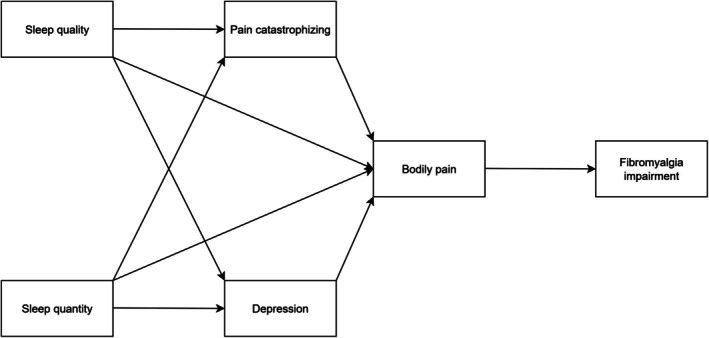
Initial model.

In the end, functional impairment in fibromyalgia (FM) emerges as a multifaceted outcome shaped by the cumulative effects of chronic pain, sleep disturbances, psychological distress and other interrelated factors. Drawing on conceptual models that describe how sleep disturbances impact central pain processing and disability (Arnison et al. 2022; Hamilton et al. 2012; Mun et al. 2020; Smith et al. 2009; Valrie et al. 2013), this study investigates a temporal pathway in which poor sleep contributes to functional impairment through elevated pain catastrophising and depressive symptoms. The hypothesised model is shown in Figure 1, with paths derived from these theoretical and empirical frameworks.

This pathway aligns with the broader clinical picture of FM, a nociplastic pain condition characterised by chronic, widespread pain, often accompanied by fatigue, sleep disturbances, cognitive impairments and depressive symptoms (Kosek 2024; Kosek et al. 2016; Sarzi‐Puttini et al. 2020). Globally, FM affects approximately 2% of the population (Heidari et al. 2017; Queiroz 2013). Although the full pathogenic mechanisms of FM remain to be elucidated (Siracusa et al. 2021), central nervous system dysfunction is evident through increased pain sensitivity and impaired pain modulation (O'Brien et al. 2018).

These abnormalities contribute to the clinical presentation, with pain severity correlating with perceived disability, though this relationship varies across subgroups, emphasising FM's heterogeneity (Turk et al. 1996). Psychological factors, including pain catastrophising and kinesiophobia, influence this relationship (Cigaran‐Mendez et al. 2022; Varallo et al. 2022), with pain‐specific cognitive biases, such as perceiving pain as a harmful threat, driving catastrophising (Crombez et al. 2012; Edwards et al. 2006; Todd et al. 2015). Compared to other chronic pain conditions, FM patients exhibit higher levels of catastrophising (Montoro and Galvez‐Sanchez 2022; Wheeler et al. 2019). Neuroimaging studies show that catastrophising modifies cerebral processing of noxious stimuli (Sandstrom et al. 2023, 2020) and increased connectivity between the posterior cingulate cortex and the salience network mediates the relationship between catastrophising and pain widespreadness (Ellingsen et al. 2021). Catastrophising is consistently associated with exaggerated perceptions of functional limitations (Estevez‐Lopez et al. 2018). It often co‐occurs with depression, which affects up to 85% of people with chronic pain (Bair et al. 2003), and is more common in FM than in pain‐free populations (Chang et al. 2015; Yepez et al. 2022). Depression partially mediates the association between pain intensity and physical functioning (Steiner et al. 2017).

Adding further complexity, sleep disturbances are also common in FM, with many patients experiencing both disrupted sleep and mood disorders (Pae et al. 2008; Roizenblatt et al. 2011). Poor sleep may trigger or intensify FM symptoms (Choy 2015), with affected individuals reporting worse sleep than controls (Wu et al. 2017). A negative correlation between sleep and severity of FM symptoms (Andrade et al. 2020; Ughreja et al. 2024) underscores the need for further research into the role of sleep disruption in FM (Choy 2015), particularly given that sleep disturbances exacerbate declines in physical functioning over time (Bigatti et al. 2008). Pain‐related cognitive biases may increase arousal, perpetuating a cycle of pain and sleep disruption (Todd et al. 2022), while depressive symptoms and pain vigilance mediate the relationship between poor sleep and pain severity (Harrison et al. 2016). Furthermore, pain, sleep disturbances and mood disorders share central nervous system pathways, highlighting their interconnected impact on quality of life (Finan and Smith 2013; Haidary et al. 2024; Spaeth et al. 2011).

Taken together, prior conceptual models highlight interconnections between sleep disturbance, psychological distress and disability in FM (Arnison et al. 2022; Burgess et al. 2019; Hamilton et al. 2012; Smith et al. 2009; Valrie et al. 2013). These frameworks, along with empirical evidence, suggest that poor sleep may contribute to pain and functional impairment both directly and indirectly by increasing pain catastrophising and depressive symptoms, two psychological processes consistently linked to disability and often occurring together (Bair et al. 2003; Chang et al. 2015; Estevez‐Lopez et al. 2018; Montoro and Galvez‐Sanchez 2022; Steiner et al. 2017). Guided by this foundation, we hypothesised that poor sleep would be associated with pain and functional impairment in FM through both direct and indirect pathways involving these psychological mediators (Figure 1).

## 2 Methods

### 2.1 Participants and Procedure

Participants were recruited through advertisements in regional daily newspapers. To increase the sample size and meet the analytical requirements for path analysis, data from two original studies on FM were combined: the *PAINOMICS* study (ClinicalTrials.gov Identifier: NCT01226784) and the *GLORIA* study (Open Science Framework registration: https://osf.io/8zqak). This resulted in a total of 253 individuals included in the present study. Both studies adhered to the principles of the Declaration of Helsinki and were approved by the Swedish Ethical Review Authority (Dnr 2010/1121‐31/3 and 2014/1604‐31/1). Written and oral information about the study was provided to all participants, and written informed consent was obtained prior to participation.

Inclusion criteria were consistent across studies and required participants to be female, of working age (20–60 years), and to meet both the 1990 and 2011 American College of Rheumatology (ACR) classification criteria for FM (Wolfe et al. 2011, 1990). While some exclusion criteria varied slightly in scope (see Supporting Information S1), both protocols excluded participants with other primary pain conditions, severe somatic or psychiatric illness, or confounding medication use, thus maintaining overall comparability in sample characteristics.

In both studies, participants were introduced to the study protocols during their first visit and completed standardised questionnaires assessing sleep, pain, psychological symptoms and functional impairment.

### 2.2 Measures

Participants completed a battery of standardised, validated self‐report questionnaires to assess sleep problems, psychological symptoms, bodily pain and functional impairment.

The Pittsburgh Sleep Quality Index (PSQI) (Buysse et al. 1989) is a 19‐item self‐report instrument that generates seven component scores and a global score assessing sleep quality over the past month. Higher global scores (0–21) indicate poorer overall sleep quality, and the PSQI has demonstrated strong reliability and validity in both clinical and non‐clinical populations (Mollayeva et al. 2016). In the present study, we used two PSQI components: subjective sleep quality (Item 6; rated 0 = very good to 3 = very bad) and sleep duration (Item 4; rated 0: > 7 h to 3: < 5 h), rather than the global score. These components were selected because they are theoretically central to our model, capture a large portion of the construct of sleep health (Buysse 2014) and provide more targeted information for interpretation and clinical implications. This approach also avoided the need for a more complex analytical strategy using all seven components. Higher scores on these components reflect poorer perceived sleep quality and shorter habitual sleep duration (Buysse et al. 1989).

The Fibromyalgia Impact Questionnaire (FIQ) (Burckhardt et al. 1991), a 20‐item measure, was used to evaluate disease‐related symptoms and disability with a total score between 0 and 100, higher scores indicating poorer health (Burckhardt et al. 1991). The FIQ is extensively validated and is considered to have good psychometric properties (Bennett 2005).

The Bodily Pain subscale of the Short Form–36 health survey (SF‐36BP) (Ware Jr. and Sherbourne 1992), was used to assess pain severity and the extent to which pain interferes with activities. Scores range from 0 to 100 (converted from raw scores), with lower scores indicating greater pain severity and more activity interference. The SF‐36BP has demonstrated acceptable psychometric properties in populations with chronic pain (LoMartire et al. 2020).

Pain catastrophising was assessed using the Pain Catastrophizing Scale (PCS) (Sullivan et al. 1995), a 13‐item questionnaire with a total score from 0 to 52. Higher scores correspond to more pain‐related catastrophising, and good psychometric properties have been found for PCS total scores (Wheeler et al. 2019).

The Hospitalised Anxiety and Depression Scale (HADS) (Zigmond and Snaith 1983) is a psychometric measure for nonpsychiatric patients and a valid and reliable measure of emotional distress in individuals with longstanding pain (LoMartire et al. 2020). In the present study, the subscale assessing depression on a 21‐point scale (HADS‐D) was used, with higher scores indicating more severe symptoms.

### 2.3 Statistical Analyses

Descriptive statistics and correlational analyses were conducted using SPSS version 27 (IBM Corp 2020). The assumptions for path analysis were evaluated following established guidelines (Barbeau et al. 2019). Missing data ranged from 3% to 13% at the scale level. Both univariate and multivariate data conformed to normal distribution (Kim 2013), with no univariate or multivariate outliers identified. Multicollinearity was not identified as an issue in the analysis. The sample size, exceeding 200 participants, was considered adequate for the analysis (Kline 2016).

The hypothesised model, depicted in Figure 1, was explored using path analyses in Mplus version 8 (Muthén and Muthén 2017). To examine mediational effects, the 95% confidence intervals (CIs) for indirect effects were computed using 5000 bootstrap resampling; if the estimated CIs included 0, it was interpreted that the proposed mediational effect was not statistically significant (Dearing and Hamilton 2006; MacKinnon 2008). Maximum likelihood (ML) estimation was used, and missing data were handled using full information ML (FIML) in Mplus. Model fit was determined using the following indices and guidelines: *χ* ^2^: *p* > 0.05; standardised root mean square residual (SRMR): < 0.08; root mean square error of approximation (RMSEA): < 0.06; comparative fit index (CFI): > 0.90 (Hu and Bentler 1999; Kline 2016). Although path analysis allows the examination of hypothesised directional relationships, the cross‐sectional design precludes conclusions about causal effects.

## 3 Results

### 3.1 Descriptive Statistics

Table 1 presents the demographic and clinical characteristics of the sample, while Table 2 presents descriptive statistics and Pearson correlation coefficients for the key study variables.

**TABLE 1:** Descriptive statistics of the 253 study participants.

	GLORIA (*n* = 111)	PAINOMICS (*n* = 142)
Age	47.1 (8.2)	51.7 (9.1)
Fibromyalgia duration (years)	10.6 (7.5)	10.6 (7.9)
Tender points (number)	16.5 (1.8)	15.7 (1.9)

**TABLE 2:** Descriptive statistics and correlations of the key variables.

	*M*	SD	1	2	3	4	5
1. Fibromyalgia impact (FIQ)	62.08	16.10					
2. Depression (HADS‐D)	7.11	3.94	0.56**				
3. Pain catastrophising (PCS)	19.19	10.89	0.39**	0.38**			
4. Sleep quality (PSQI)	1.97	0.80	0.46**	0.37**	0.28**		
5. Sleep quantity (PSQI)	1.35	1.10	0.19**	0.20**	0.20**	0.55**	
6. Bodily pain (SF‐36)	32.53	14.50	−0.64**	−0.40**	−0.40**	−0.41**	−0.18*

Abbreviations: FIQ = Fibromyalgia Impact Questionnaire; HADS‐D = Hospital Anxiety and Depression Scale‐depression subscale; PCS = Pain Catastrophizing Scale; PSQI = Pittsburgh Sleep Quality Index; SF‐36 = Short Form Health Survey‐36.

^*^ *p* < 0.05.

^**^ *p* < 0.01.

### 3.2 Path Analyses

The hypothesised model, depicted in Figure 1, was tested using path analysis. The model evaluated the direct and indirect relationships among sleep quality and quantity, pain catastrophising, depressive symptoms, bodily pain and functional impairment. The fit of this model was not acceptable, *χ* ^2^ (5, *N* = 241) = 99.95, *p* < 0.001, CFI = 0.276, RMSEA = 0.301, 90% CI = [0.252–0.354], SRMR = 0.159. Given the poor fit of the initial model, theory‐driven modifications were made based on modification indices using ML with robust standard errors (MLR) to improve the model fit. In the first step, a direct path from the HADS‐D to the FIQ was entered, supported by evidence that depression independently predicts disability in FM and is central in biopsychosocial pain models (Chang et al. 2015; Hamilton et al. 2012; Steiner et al. 2017). Model fit remained poor (*χ* ^2^ (4, *N* = 241) = 42.31, *p* < 0.001, CFI = 0.713, RMSEA = 0.212, 90% CI = [0.167–0.281], SRMR = 0.104). In the second step, a path from the PCS to the FIQ was used, in line with cognitive‐emotional models positioning catastrophising as a direct determinant of disability independent of pain intensity (Lopez‐Gomez et al. 2023; Montoro and Galvez‐Sanchez 2022). This improved but did not optimise model fit (*χ* ^2^ (3, *N* = 241) = 6.35, *p* = 0.04, CFI = 0.972, RMSEA = 0.108, 90% CI = [0.023–0.206], SRMR = 0.031). Finally, a path from sleep quantity (PSQI component) to FIQ was included. Although sleep quality often shows stronger associations with impairment, shorter sleep duration is also linked to poorer physical functioning, fatigue and symptom severity in FM, consistent with multidimensional sleep health frameworks (Bigatti et al. 2008; Buysse 2014; Ughreja et al. 2024). This yielded acceptable fit (*χ* ^2^ (2, *N* = 241) = 2.28, *p* = 0.13, CFI = 0.990, RMSEA = 0.052, 90% CI = [0.010–0.128], SRMR = 0.017).

One sensitivity analysis was carried out when two of the FIQ items (Number 14 assessing pain and Number 19 determining depression) were removed from the FIQ total sum variable. This analysis was executed since the two respective FIQ items might bias the results because two similar constructs are part of the structural model, in those cases assessed by the SF‐36BP (bodily pain) and the HADS‐D (depression). In the analysis, using the modified final model identified above and removing Items 14 and 19 in the FIQ, all fit indices suggested an acceptable model (*χ* ^2^ (2, *N* = 241) = 2.74, *p* = 0.10, CFI = 0.984, RMSEA = 0.058, 90% CI = [< 0.001–0.132], SRMR = 0.020).

**FIGURE 2:**
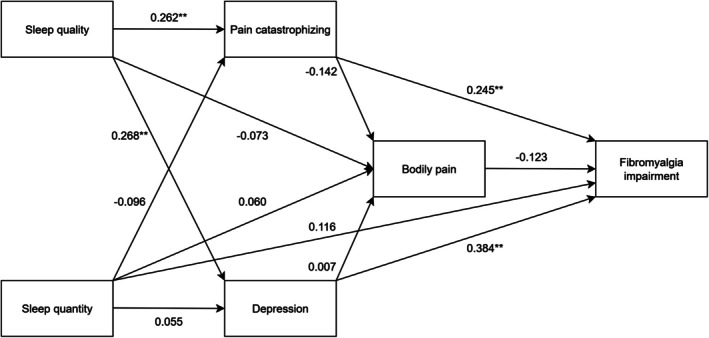
Modified, final model.

The final model is depicted in Figure 2. Since the sensitivity analysis excluding two FIQ items produced results comparable to the model including them, all FIQ items were retained in the final path analysis. This final analysis revealed four significant direct effects, while none of the explored indirect effects reached significance. First, two significant direct effects concerned sleep quality from the PSQI; sleep quality displayed a significant association with pain catastrophising (*β* = 0.262, 95% CI = 0.126–0.380, *p* < 0.001) and depression (*β* = 0.268, 95% CI = 0.143–0.381, *p* < 0.001). Second, two direct effects involved two parameters being significantly related to the FIQ: pain catastrophising (*β* = 0.245, 95% CI = 0.108–0.366, *p* < 0.001) and depression (*β* = 0.384, 95% CI = 0.249–0.485, *p* < 0.001). None of the indirect effects were significant (sleep quality to FM impairment: *β* = 0.009, 95% CI = −0.004 to 0.044; sleep quality to bodily pain: *β* = −0.035, 95% CI = −0.094 to 0.012; sleep quantity to FM impairment: *β* = −0.008, 95% CI = −0.043 to 0.007; sleep quantity to bodily pain: *β* = 0.014, 95% CI = −0.008 to 0.045). The final model explained 32.5% of the variance in functional impairment, as well as 8.0% in depression, 6.9% in pain catastrophising and 3.3% in bodily pain.

## 4 Discussion and Conclusions

The present study investigated a model hypothesising that sleep difficulties contribute to altered pain processing and functional impairment in FM through psychological mechanisms. Using structural equation modelling, we examined the interrelations among sleep quality and quantity, pain intensity, pain catastrophising, depression and overall functional impairment. The findings demonstrated that poor sleep quality was directly associated with greater levels of pain catastrophising and depression, both of which, in turn, were linked to increased functional impairment. The model accounts for nearly one‐third of the variability in overall impairment.

While sleep‐related and psychological variables emerged as significant contributors to functional impairment, pain severity did not show a direct association with impairment in this model. This is noteworthy given that pain intensity has traditionally been recognised as a primary determinant of impairment in FM, showing significant correlations across nearly all dimensions of the FIQ (Campos and Vazquez Rodriguez 2012). This apparent discrepancy may reflect a more nuanced interplay, in which pain severity exerts its influence through psychological mediators, supporting a growing recognition that perceived disability in FM may be more closely linked to the cognitive and affective dimensions of pain rather than its sensory intensity alone. In line with this view, pain catastrophising and depression have emerged as key mediators in the relationship between clinical pain and functional capacity, underscoring their critical role in pain‐related disability in FM (Montoro and Galvez‐Sanchez 2022).

These findings are further echoed in the literature. A study among older adults with chronic pain highlighted that sleep quality is a key mediator in the indirect effect of pain catastrophising on depression (Lee et al. 2020). In a study of women with FM, Liedberg et al. differentiated two subgroups based on variables related to sleep quality. In line with the current study, the subgroup with lower perceived sleep quality reported a significantly greater impact of FM on their daily living, as assessed using the FIQ. This subgroup also reported significantly higher levels of catastrophising (Liedberg et al. 2015). In qualitative research, the substantial impact of poor self‐perceived sleep quality on quality of life has also been emphasised in a metasynthesis (Climent‐Sanz et al. 2020).

Although prior studies have suggested that sleep disturbances may influence pain processing and thereby contribute to functional impairment, our analyses did not provide empirical support for this pathway. While chronic insufficient sleep may impair central pain‐modulatory processes (Simpson et al. 2018), misestimation of sleep duration is common among FM patients (Okifuji and Hare 2011; Stuifbergen et al. 2010). Self‐reported questionnaires might not reliably assess objective sleep duration (Hinchado et al. 2023; Wu et al. 2017), and the PSQI captures dimensions that differ from those assessed by actigraphy and polysomnography (PSG) (Buysse et al. 2008). These discrepancies complicate efforts to detect robust associations between objective sleep measures and clinical outcomes. Another explanatory perspective considers that a significant subset of FM patients may experience pain driven by abnormalities in the peripheral nervous system and/or autoimmune mechanisms (Evdokimov et al. 2019; Kosek 2024; Krock et al. 2023), which might be less influenced by sleep disturbances compared to alterations in central pain modulation.

Although the present study identifies direct and indirect relationships between variables, it does not explore intermediary mechanisms, such as central sensitisation (CS), in depth. This is a notable omission, as CS is increasingly recognised as a fundamental mechanism in underlying a range of nociplastic pain conditions, including FM. CS refers to an amplification of neural signalling within the central nervous system that results in hypersensitivity to pain and other sensory stimuli, with evidence suggesting that CS may not only contribute to symptom severity but may also precede the development of certain symptoms, playing a potential causal role in the development of functional impairment (Sluka and Clauw 2016). In addition to CS, the role of neuroinflammatory processes and stress‐related pathways warrants attention. Neuroinflammation, as evidenced by increased glial activation in the brain (Albrecht et al. 2019) and elevated cerebrospinal fluid interleukin‐8 levels (Kadetoff et al. 2012), is known to play a critical role in FM. Additionally, the involvement of stress‐related pathways, including dysregulation of the sympathetic nervous system and the hypothalamic–pituitary–adrenal (HPA) axis, has been demonstrated in FM patients (Kadetoff and Kosek 2010), further underscoring the complex aetiology of this condition.

From a theoretical standpoint, the Sleep and Pain Diathesis (SAPD) model posits that sleep is a primary etiological factor contributing to a wide range of FM symptoms. According to this model, FM patients with more disrupted sleep report higher levels of both psychological symptoms and physical disability, with the link between sleep and disability being mediated by pain (Hamilton et al. 2012). While the current study supports the SAPD model, it does not confirm the specific influence of pain, also proposed in the conceptual working model of Smith et al. (2009). Finally, the findings of the present study are partly consistent with the Intraday Process Model (Mun et al. 2020), which posits that both nonrestorative sleep and morning pain catastrophising have direct associations with end‐of‐day activity interference. Taken together, these findings highlight the multifactorial nature of FM impairment and the importance of targeting both sleep quality and psychological factors in treatment.

The current study has some strengths and limitations. The methodology of this study, which involved iterative adjustments to the model and sensitivity analyses, aligns with the necessity of employing rigorous statistical techniques to accurately capture and interpret the complex interplay of factors in chronic health conditions. This approach ensures that theoretical constructs are adequately represented in empirical models. The use of modification indices to improve model fit involves making data‐driven adjustments to the theoretical model based on statistical recommendations. While this approach can enhance the model's alignment with the specific dataset, it should be applied cautiously and always supported by theoretical justification. The study's cross‐sectional design prevents the establishment of causal relationships, and the reliance on self‐reported data for sleep may introduce bias due to subjective interpretation. The absence of an objective measure of sleep constrains the study's capacity to thoroughly examine the proposed pathway that sleep disturbances contribute to pain processing and subsequent functional impairment. An ongoing study by our group utilising PSG in FM patients and healthy controls aims to investigate the relationship between sleep, psychological distress and impairment in FM, providing more objective assessments of total sleep time and sleep quality. Additionally, the study focused on specific pathways and variables, potentially overlooking other relevant factors that could influence FM impairment, such as other psychological factors, medication use, comorbid conditions, social support, lifestyle factors and environmental influences. Participants were recruited through newspaper advertisements, a community‐based approach that broadened outreach beyond clinical settings and reduced referral bias by capturing individuals representative of the general FM population. The inclusion of only female participants aged 20–60 years allowed for a focused analysis of FM mechanisms in the population most affected by the condition, minimising variability related to sex‐ and age‐specific differences in pain perception, hormonal influences and psychosocial factors. However, these criteria also limit the generalisability of the findings. Future studies are warranted to determine whether the observed relationships extend to male patients and individuals outside this age range, and to explore potential sex‐ and age‐related differences in FM presentation and mechanisms. Data were drawn from two studies with shared inclusion criteria and identical self‐report measures. One study applied stricter exclusion criteria due to neuroimaging protocols, which may have influenced comorbidity profiles. As all data were collected under comparable conditions using the same instruments, any impact on the current findings is likely minimal.

Sleep quality is closely associated with FM symptoms (Andrade et al. 2020). The results of the current study emphasise the pivotal role of subjective sleep quality in the manifestation of FM impairment and suggest mechanisms that may influence this condition. The significance of incorporating interventions that improve sleep quality into chronic pain management has been emphasised in a meta‐analysis of randomised controlled trials, highlighting their potential to enhance overall health outcomes. This analysis demonstrated that cognitive‐behavioural therapy for insomnia (CBT‐i) leads to substantial improvements in sleep and pain at post‐treatment (Selvanathan et al. 2021). Another meta‐analysis evaluated the effectiveness of CBT‐i in individuals with FM compared to other non‐pharmacological treatments, revealing significant improvements in sleep quality, pain, anxiety and depression, although the evidence quality is low (Climent‐Sanz et al. 2022). In a study comparing cognitive‐behavioural therapy focused on pain with a combined cognitive‐behavioural therapy focused on both pain and insomnia for FM patients, the therapy focused solely on pain increased time in bed and total sleep time. In contrast, the combined therapy resulted in more significant improvements in sleep quality, including higher sleep efficiency and longer duration of deep sleep (Prados et al. 2020). Additionally, sleep restriction therapy (SRT), a key component of CBT‐i, has been shown to significantly improve sleep quality metrics like insomnia severity, sleep efficiency, sleep onset latency and wake time after sleep onset, without affecting total sleep time (Maurer et al. 2021). This highlights the importance of prioritising sleep quality over sleep quantity for improving overall well‐being. Recent evidence suggests that sleep quality is a robust predictor of quality of life, whereas sleep quantity has a more limited effect (Kudrnacova and Kudrnac 2023). These findings emphasise the need for clinical interventions that focus on enhancing sleep quality as a central component of holistic FM management strategies.

## Author Contributions

**Kristoffer Bothelius:** conceptualization, writing – original draft, methodology, project administration, writing – review and editing, formal analysis. **Eva Kosek:** investigation, funding acquisition, writing – review and editing, resources. **Markus Jansson‐Fröjmark:** conceptualization, methodology, formal analysis, data curation, writing – review and editing, visualization, writing – original draft.

## Conflicts of Interest

The authors declare no conflicts of interest.

## Supporting information


**Data S1:** Supporting Information.

## Data Availability

The data that support the findings of this study are available from the corresponding author upon reasonable request.
