# Supplementary material for: In the End: Associations Between Sleep Disturbance and Functional Impairment in Fibromyalgia—A Path Analysis Study
Source: J Sleep Res. 2025 Sep 17;35(3):e70199. doi: 10.1111/jsr.70199 (PMC13193491; doi:10.1111/jsr.70199)
Supplement: Supplementary file 1 — Data S1: Supporting Information. [file JSR-35-e70199-s001.docx]

**Supplementary Information**

*Detailed information on the exclusion criteria.*

I. “PAINOMICS”, 2010/1121-31/3 (ClinicalTrials.gov Identifier: NCT01226784)

Participants were excluded from the PAINOMICS study based on the following criteria:

- Systolic blood pressure exceeding 160 mmHg or diastolic blood pressure exceeding 90 mmHg.
- Presence of osteoarthritis in hip or knee joints.
- Diagnosis of severe somatic or psychiatric disorders.
- Primary causes of pain other than fibromyalgia syndrome (FMS).
- High alcohol consumption, defined as an Audit score greater than 6.

II. “GLORIA”, 2014/1604-31/1 (see study plan https://osf.io/8zqak)

For the GLORIA study, individuals were excluded if they met any of the following conditions:

- Presence of dominant pain conditions other than FMS.
- Diagnosis of rheumatic or autoimmune diseases.
- Presence of severe somatic diseases, including but not limited to neurological, cardiovascular, or cancer conditions.
- Psychiatric disorders, particularly if undergoing treatment for depression or anxiety.
- History of substance abuse.
- Current pregnancy status.
- Presence of magnetic implants.
- History of brain or heart surgery.
- Hypertension with readings greater than 160/90 mmHg.
- Obesity, defined as a body mass index (BMI) greater than 35.
- Smoking habits exceeding 5 cigarettes per day.
- Current medication regimen includes antidepressants or anticonvulsants.

Additional specific exclusions include the inability to speak or understand Swedish, self-reported claustrophobia, and the requirement to refrain from NSAIDs, analgesics, or hypnotics for at least 48 hours prior to study participation, extending to 72 hours before the second visit.
